# C‐Terminal Hsp90 Inhibitors Overcome MEK and BRAF Inhibitor Resistance in Melanoma

**DOI:** 10.1111/jcmm.70489

**Published:** 2025-03-26

**Authors:** Chitra Subramanian, Katie K. Hohenberger, Ang Zuo, Eric Cousineau, Brian Blagg, Mark Cohen

**Affiliations:** ^1^ Department of Surgery, and Biomedical and Translational Sciences Carle Illinois College of Medicine at the University of Illinois Urbana Champaign Urbana Illinois USA; ^2^ Department of Otolaryngology Stanford Medicine Stanford California USA; ^3^ Department of Pharmacology University of Notre Dame Notre Dame Indiana USA; ^4^ Beckman Institute for Advanced Science and Technology University of Illinois Urbana‐Champaign Urbana Illinois USA

**Keywords:** BRAF and MEK inhibitor, cell cycle, C‐terminal Hsp90 inhibitor KU757, oxidative phosphorylation

## Abstract

Targeted therapies for melanoma MEK and BRAF inhibitors fail due to the development of chemoresistance. As Hsp90 inhibitors target client proteins of resistant pathways, we hypothesised that C‐terminal Hsp90 inhibitors will target BRAF/MEK inhibitor resistant melanoma cells by overcoming the resistant pathways. Two melanoma cell lines, A375 and A375 MEK/BRAF inhibitor resistant (A375MEKi) were utilised. The inhibitory concentrations (IC_50_) of two C‐terminal Hsp90 inhibitors, KU757 and KU758, were determined by CellTiter Glo. RNA sequencing was performed after treatment with KU757. Pathways targeted by differentially expressed genes were evaluated by David, IPA, GSEA, and by evaluating the cell cycle, apoptosis and oxidative phosphorylation. Expression levels of hub genes were evaluated using Xena and validated by RT‐PCR. The survival analysis was performed using UALCAN. A375MEKi was not resistant to the C‐terminal Hsp90 inhibitor with a KU757 IC_50_ of 0.59 μM versus 0.64 μM and a KU758 IC_50_ of 0.89 μM versus 0.93 μM in A375 versus A375MEKi, respectively. RNA sequencing analysis revealed KU757 upregulates cell cycle checkpoint regulation and apoptosis and downregulates genes involved in the peroxisome, AKT/PI3K/MTOR, EIF2, fatty acid metabolism and oxidative phosphorylation. These pathways were further validated through survival analysis that demonstrated potential survival benefit in patients with dysregulated NDUFA7, CDC20, CDC25C, CDK1, VDAC2, HEATR5a, COL4A4, FLT3LG, BMP2, PRKCH and ADMST9. Melanomas often develop concurrent resistance to BRAF and MEK inhibitors. C‐terminal Hsp90 inhibition with KU757 appears to overcome these chemo‐resistance pathways in vitro, downregulating metabolic pathways including oxidative phosphorylation and the cell cycle, warranting further in vivo translation. The novel C‐terminal HSP90 inhibitor KU757 effectively targets primary and BRAF and MEK inhibitor‐resistant melanoma cells equally by affecting oxidative phosphorylation and the cell cycle.

## 1 Introduction

The incidence of new invasive melanomas diagnosed in the United States annually is increasing at a rate of approximately 16% for the past 5 years [1]. Recent estimates suggest one in 28 men and one in 41 women will develop invasive melanoma in their lifetime [2]. While local disease is often amenable to surgical resection and carries a more favourable survival rate, regional (stage III) and distantly metastatic (stage IV) disease carry a less‐favourable prognosis, with 5‐year survival rates estimated at 63.6% and 22.5%, respectively [3]. While sentinel lymph node biopsy helps identify patients with occult nodal disease and confers some treatment benefit, several landmark studies, including the MSLTII and DeCOG‐SLT trials, have failed to demonstrate a significant survival advantage for patients undergoing completion lymph node dissection [4, 5, 6, 7]. The mainstay of therapy for these patients as well as those with gross regional and metastatic disease has shifted to targeted chemotherapy and immunotherapy [8, 9].

Genetically, melanomas represent a heterogeneous population of tumours with genomic aberrations most commonly affecting NRAS (15%–20%) and BRAF (40%–50%) [10]. NRAS and BRAF are both oncogenes leading to constitutive activation of the mitogen‐activated protein kinase (MAPK) pathway [11]. Targeted combination therapy with BRAF and MEK inhibitors is currently utilised in mutant BRAF tumours with or without immunotherapies [12]. Therapeutic combinations include dabrafenib plus trametinib, vemurafenib plus cobimetinib and encorafenib plus binimetinib, with each demonstrating an advantage over vemurafenib alone; but unfortunately, the effect is not durable, with short progression‐free survival intervals of 11.1, 12.3 and 14.9 months, respectively [13, 14, 15]. All currently approved combinations are hallmarked by the development of drug resistance, with 5‐year progression‐free survival estimated at less than 20%, leaving many patients with dual BRAF inhibitor (BRAFi) and MEK inhibitor (MEKi) resistant tumours and limited therapeutic options.

Resistance to therapies targeting the MAPK pathway occurs not only through further activation of the MAPK pathway but also through alternative pathways [16]. These include dysregulation of PI3/AKT/MTOR signalling, cell adhesion, cell cycle dysregulation and aberrant PDGFRB, AXL and EGFR signalling [17]. Given the multifactorial nature of this resistance, the use of Hsp90 inhibitors has long presented an appealing alternative therapeutic option in melanoma [18]. Hsp90 acts as a master chaperone with client proteins regulating oncogenic processes in all 10 of Weinberg's hallmarks of cancer [19]. Direct Hsp90 chaperone effects are known to act on the MAPK pathway (BRAF and MEK are clients of Hsp90), as well as the PI3K pathway, NF‐KB signalling, WNT signalling and the unfolded protein response, as all contain client proteins chaperoned by Hsp90 [20]. Prior work has demonstrated cytotoxic and cytostatic effects of Hsp90 inhibitors in melanoma and even the ability to delay resistance to BRAF and MEK inhibitors [21, 22, 23].

Despite multiple clinical trials, no Hsp90 inhibitors to date have received FDA approval for use in melanoma. The majority of the Hsp90 compounds that have been tested in clinical trials target the N‐terminal ATP binding site of Hsp90 and have demonstrated dose‐limiting toxicities and lack of durable efficacy in clinical trials. This failure in the clinic has been shown in part to be due to the nonspecific pan‐inhibition of Hsp90, which is known to activate the pro‐survival heat shock response with upregulation of Hsp70 and its pro‐survival effects leading to the need for higher doses of Hsp90 inhibition to maintain efficacy [24]. This heat shock response induction is a key driver leading to the dose escalation and DLTs observed in the trials. To overcome these limiting issues of N‐terminal Hsp90 inhibitors, our group was the first to develop C‐terminal Hsp90 inhibitors which selectively block certain chaperone functions of Hsp90 including dysregulating many oncogenic client protein kinases without upregulating the cytoprotective heat shock response [25, 26]. Previously, our group has demonstrated in vitro synergistic effects when a C‐terminal Hsp90 inhibitor is combined with either BRAF or MEK inhibitors [27, 28]. The response to Hsp90 inhibitors in tumours that have already developed drug resistance after treatment with MAPK and/or BRAF inhibitors has not been thoroughly explored. Therefore, in the present study, we evaluated the efficacy of two novel C‐terminal inhibitors in targeting chemo‐resistance pathways in melanoma cells resistant to BRAF‐ and MEK‐inhibitors. We then performed RNA‐seq analysis to identify the pathways that are altered after treatment with the novel C‐terminal Hsp90 inhibitor KU757. Finally, we performed a multi‐genomic database analysis to identify novel hub genes associated with KU757 treatment that correlate with melanoma patient survival.

## 2 Methods

### 2.1 Cell Lines and Reagents

Validated melanoma cell lines A375, which contain the BRAF^V600E^ mutation, and an A375 MEK inhibitor‐resistant line (A375MEKi) were purchased from ATCC (Manassas, VA). The A375MEKi cell line was generated from the A375 parent with the MEK1^Q58P^ knock‐in mutation generated by CRISPR as previously described [29]. A375 and A375MEKi cells were cultured in Dulbecco's Modified Eagle Media (DMEM) (Thermo Fisher Scientific, Waltham, MA) supplemented with 10% fetal bovine serum (Sigma‐Aldrich, St. Louis, MO) and 1% penicillin/streptomycin (Sigma‐Aldrich). Cells were maintained in T75 flasks (Thermo Fischer Scientific, Waltham, MA) at 37°C and 5% CO_2_. Cells were passaged in 0.25% trypsin–EDTA (Sigma‐Aldrich) when indicated, with experimental utilisation between passages 3–8. Drugs utilised included the BRAF inhibitor vemurafenib (Selleck Chemicals, Houston, TX) and the MEK inhibitor cobimetinib (AdooQ Bioscience, Irvine, CA). C‐terminal Hsp90 inhibitors, including KU757 and KU758 (structure shown in Figure S1) were provided courtesy of Brian Blagg (University of Notre Dame, Indiana, IN) and developed from novobiocin‐based analogs as previously described [30].

### 2.2 Cell Viability Assay

Cell viability in response to drug treatment was assessed by CellTiter Glo luminescent Cell Viability Assay (Promega, Madison WI). Briefly, A375 or A375MEKi cells were plated at a density of 1500 cells per well in a 96‐well black‐walled, clear‐bottom plate (Thermo Fisher Scientific) and cultured overnight. Baseline ATP expression was assessed at 24 h with luminescence read on a BioTek Synergy Neo plate reader with Gen 5 software (BioTek, Winookski, VT). Remaining cells were then treated with serial dilutions of cobimetinib starting from 1 μM, vemurafenib starting from 40 μM, KU757, KU758 starting from 10 μM, or DMSO control for 72 h, and the CellTiter Glo assay was repeated. Dose response curves and half‐maximal inhibitory concentration (IC_50_) were calculated using GraphPad Prism 9.1.2 Software (San Diego, CA). Drug treatments were done in triplicate, and the values were presented as mean ± SD.

### 2.3 RNA Sequencing and Pathway Analysis

A375 or A375MEKi were seeded in 10 cm plates and grown to 80% confluence under culture conditions previously described. Cells were treated with IC_50_ concentration of KU757 or DMSO control in duplicate. RNA was isolated using RNeasy Micro Kit (QIAGEN, Hilden Germany) with on column DNase digest with RNase Free DNase Kit (Qiagen). RNA integrity was verified, and sample concentrations were normalised using the Nanodrop Bioanalyzer (Thermo Fisher). RNAseq was carried out using Illumina Novaseq after verifying quality control at the University of Michigan Advanced Genomic Core. Fastq files were converted to raw counts using Deseq2 in R program. Qiagen Gene Globe RNA analysis portal was used to identify differentially expressed genes and top targeted pathways using Qiagen Ingenuity Pathway (IPA). Fold change greater than 2 with a Bonferroni adjusted *p* < 0.05 was considered statistically significant.

### 2.4 Enrichment Analysis of Differentially Expressed Genes

Kyoto KEGG pathway and Gene Ontology (GO) enrichment pathways were used to evaluate the pathways that are enriched by differentially expressed genes after treatment with our C‐terminal inhibitor KU757. Additionally, Gene Set Enrichment Analysis (GSEA) was used to verify the up‐and down‐regulated pathway genes associated with MEK/BRAF inhibitor resistance as well as KU757's effect on targeting the resistant pathways. Ingenuity Pathway (IPA) was further used to identify upstream regulators altered by differentially expressed genes.

### 2.5 Identification, Validation and Survival Analysis of Hub Genes

Hub genes that are targeted by our novel compound KU757 were identified from genes belonging to resistance pathways that are targeted by our drug using a protein–protein interaction network in the STRING database in Cytoscape. In addition, differentially regulated genes were assessed for their impact on survival. XENA was used to validate the differential expression in The Cancer Genome Atlas (TCGA) dataset, which included 470 melanoma patient samples. Finally, the impact of differential regulation on overall survival was assessed both by genes and by pathways using Kaplan– Meier Survival analysis with Bonferroni correction for multiple comparisons, with significance defined as *p* < 0.05.

#### 2.5.1 Measurement of Oxidative Phosphorylation (OXPHOS) Using Seahorse Analyzer

Mitochondrial OXPHOS was measured using a Seahorse XFe96 analyzer (Agilant Technologies, Santa Clara, CA). Melanoma cells A375 and A375 MEK were seeded in a Seahorse 96‐well plate with DMEM medium and incubated overnight. The cells were then treated with 0.5 and 1 μM KU757 for 24 h, after which a mitostress assay was performed according to the manufacturer's protocol. The data were normalised to the total cell number by measuring cell viability using CellTiter‐Glo as described above in the cell viability assay.

#### 2.5.2 RT‐PCR and Western Blot Analysis

Melanoma cell lines A375 parent (A375P) and A375 MEK were treated with varying concentrations of KU757 for western blot and IC_50_ concentrations of KU757 for RT‐PCR for 24 h. After treatment, the cells were collected, and western blotting was performed to measure PARP levels, which serve as an indicator of apoptosis induction, as previously described [31]. Actin was utilised as a loading control. For RT‐PCR, total RNA was isolated and reverse transcribed. Quantitative PCR was then conducted using SYBR Green master mix along with gene‐specific primers.

#### 2.5.3 Cellcycle Analysis

Melanoma cells grown to 60%–80% confluence were treated with IC_50_ concentrations of KU757 for 24 h. After treatment, the cells were harvested, fixed with 70% ethanol and stored at −20°C until analysis. The samples were then centrifuged, and the cell pellet was stained with propidium iodide (PI) solution (40 μg/mL PI and 100 mg/mL RNase). The stained cells were incubated at 37°C for 30 min before undergoing cell cycle analysis using a BD FACSymphony A1 system (BD Biosciences, San Jose, CA) at the University of Illinois Urbana‐Champaign Flow Cytometry Core. Each experiment was conducted in triplicate, and only viable cells without DNA fragmentation were analysed using FCS Express software (De Novo Software, Pasadena, CA).

## 3 Results

### 3.1 C‐Terminal Hsp90 Inhibitors Target BRAF/MEK Inhibitor‐Resistant Melanoma Cells

**FIGURE 1:**
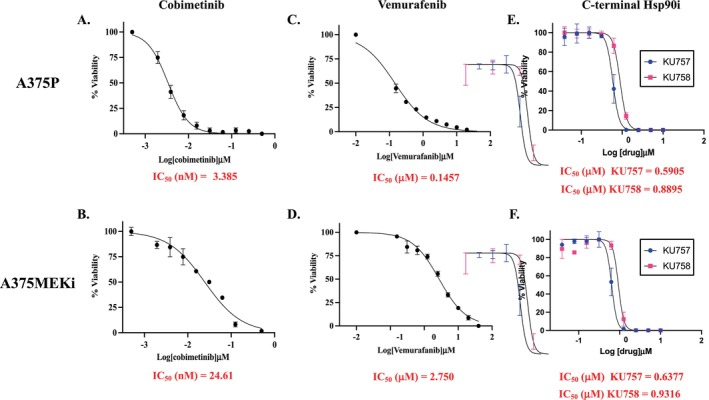
A375 and A375MEKI cell viability curves. Dose response curves demonstrate 72 h cell viability versus treatment dose as assessed by CellTiter Glo. A375 cells (A) were sensitive to cobimetinib relative to A375MEKi(B). Similarly, A 375P(C) was sensitive to vemurafenib relative to A373MEKi(D). A375 (E) and A375MEKi (F) were equally sensitive to KU757 and KU758.

Despite advances in the treatment of melanoma with BRAF and MEK inhibitor combinations and immune checkpoint inhibitors, the development of resistance is very common. Therefore, we first evaluated the efficacy of our C‐terminal Hsp90 inhibitors for targeting both A375 parent cells and BRAF/MEK inhibitor‐resistant cells. A375 and A375MEKi cell lines were validated for resistance to the MEK inhibitor cobimetinib and the BRAF inhibitor vemurafenib (Figure 1A–D). The inhibitory concentration of cobimetinib was 3.39 nM [95% CI 3.2–3.6 nM] versus 24.6 nM [95% CI: 20.8–29.0 nm] for A375 and A375MEKi, respectively, consistent with MEK inhibitor resistance (> 5 fold resistance compared to parent line). Similarly, the IC_50_ for vemurafenib was 0.146uM [95% CI: 0.125–0.168 μM] versus 2.76 μM [95% CI: 2.481–3.046 μM] for A375 versus A375MEKi, respectively. Next, cell viability in response to KU757 and KU758 treatment was assessed. MEK resistance had no effect on sensitivity to C‐terminal Hsp90 inhibitors, and A375 and A375MEKi cells were equally sensitive to KU757 and KU758 (Figure 1E,F). The IC_50_ was 0.59 μM versus 0.63 μM [95% CI 0.5516–0.6220 and 0.6133–0.6731] for KU757 and 0.89 μM versus 0.93 μM for KU758 [95% CI 0.8465–0.9348 and 0.8295–1.03] in A375 versus A375MEKi, respectively.

### 3.2 RNAseq Analysis to Identify Target Genes and Resistant Pathways After Treatment With KU757

**FIGURE 2:**
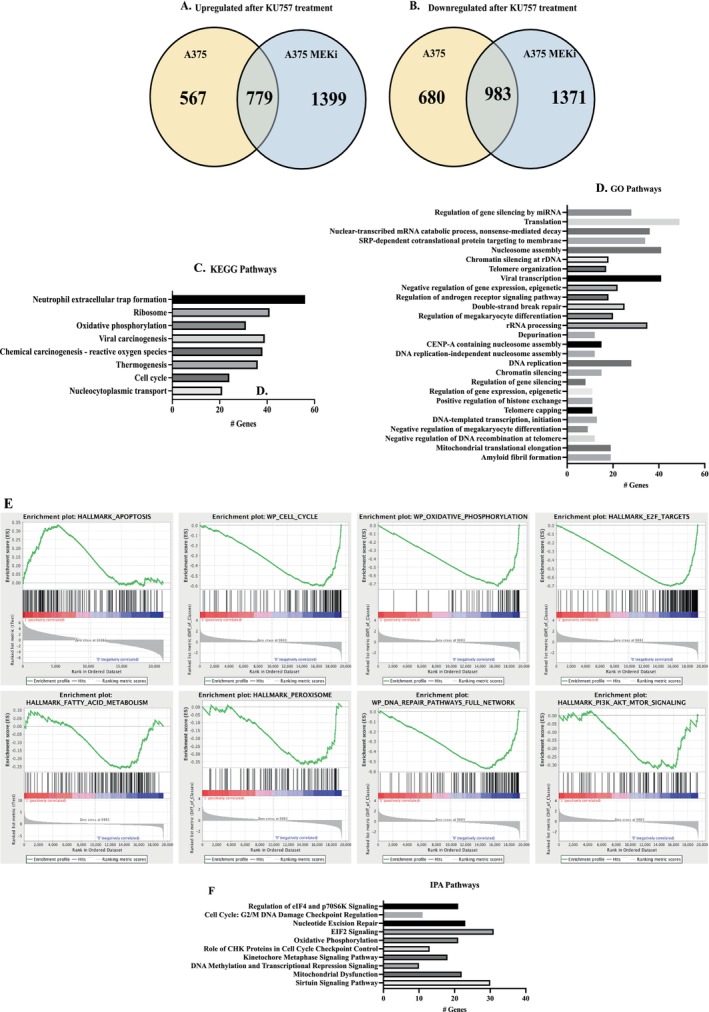
Venn Diagram of differentially expressed mRNA transcripts after KU757 treatment. (A) RNAseq revealed 779 genes commonly upregulated in both A375 and A375MEKi following KU757 treatment. (B) mRNA expression of 983 genes was downregulated in both A375 and A375MEKi cells after KU757 treatment. KEGG(C), GO(D), GSEA(E) and IPA(F) analyses reveal pathways dysregulated by KU757 in A375MEKi cells. KEGG, GO and IPA charts represent the number of significantly differentially regulated genes within each pathway. The GSEA figure indicates the directional fold change for each gene within the listed pathway (*y*‐axis) arranged by the strength of correlation with pathway regulation (*x*‐axis). Overall, these data suggest that cell cycle and oxidative phosphorylation are most significantly affected following KU757 treatment.

Given increased cell line sensitivity to KU757 relative to KU758, RNA sequencing was performed on A375 and A375MEKi melanoma cells after treatment with IC_50_ concentrations of KU757 or DMSO control. mRNA expression values were obtained for 19,933 genes. Those genes that were either significantly upregulated or downregulated both in A375 and A375MEKi following KU757 treatment were identified. 779 mRNA transcript levels were upregulated both in A375 and A375MEKi. Similarly, 983 mRNA transcripts were downregulated in both cell lines following KU757 treatment (Figure 2A,B). To evaluate resistant pathways that are altered by KU757 treatment, the DAVID database was utilised to perform KEGG pathway analysis (Figure 2C). The analysis revealed alterations in several oncogenic pathways including neutrophil extracellular trap formation, ribosome components, viral carcinogenesis, reactive oxygen species (ROS), thermogenesis, oxidative phosphorylation, cell cycle and nucleocytoplasmic transport after treatment with KU757. GO enrichment analysis was performed to identify key cellular processes that are altered by KU757, as shown in Figure 2D. Gene Set Enrichment analysis (GSEA) of upregulated and downregulated genes revealed upregulation of genes involved in the cell cycle checkpoint and the apoptotic pathway and downregulation of genes involved in the peroxisome, AKT/PI3K/MTOR, EIF2 and fatty acid metabolism and oxidative phosphorylation (Figure 2E).

Qiagen Ingenuity pathway analysis has a novel function to identify upstream regulators and transcription factors of differentially expressed genes in addition to identifying altered pathways. Therefore, we performed IPA of previously identified differentially regulated genes with a significant Bonferroni adjusted *p* < 0.05. Analysis validated significant (*p* < 0.001) downregulation of oxidative phosphorylation, EIF2 signalling, G2/M DNA Checkpoint Regulation, Kinetochore Metaphase signalling, CHK protein in the cell cycle and nucleotide excision after KU757 treatment, and identified several upstream regulators (Figure 2E). The top upstream regulators inhibited upon KU757 treatment (Table 1) include DAP3, ALKBH1, NSUN3, CKAP2L and the E2F group (*p* = 1.58e^−16^, 5.52e^−12^, 5.52e^−12^, 5.69e^−14^, 1.06e^−16^). ALKBH1 and NSUN are RNA modifiers that regulate cell fate, and E2F is upregulated in several cancers as it plays an important role in the cell cycle. Upregulation of DAP3 and CKAP2L are also known oncogenes that promote cancer development and resistance to radiation. The main upstream activators after treatment with KU757 include the cytokine TNF, NfkB1‐RelA, E2F and CHD1 (*p* = 1.46e^−^11, 1.17e^−11^, 4.18e^−20^ and 3.24e^−12^). Upregulation of the TNF cytokine and NfkB1‐RelA indicates necroptotic cell death as the apoptotic pathway, consistent with the apoptosis upregulation identified in GSEA analysis after KU757 treatment. Overall, these results support that KU757 overcomes some of the pathways driving BRAF and MEK inhibitor resistance by downregulating key tumour‐promoting genes and upregulating tumour suppressors, several of which are drivers of MAPK resistance. In addition to the top oncogenic pathways that were targeted, we also assessed the impact on select metabolic enzymes including LDH, MDH and SHMT as well as the expression of heat shock proteins including Hsp70, Hsp32 and HSF‐1 and noted that mRNA copy number was decreased following KU757 treatment in A375MEKi cells.

**TABLE 1:** Upstream regulators of differentially regulated genes after treatment of melanoma cells with KU757 in IPA.

Upstream regulator	Prediction	*Z*‐score	Number of targets	*p*	−log_10_ of *p*
DAP3	Inhibited	−3.464	12	1.58 e^−16^	15.801
ALKBH1	Inhibited	−2.828	8	5.52 e^−12^	11.258
NSUN3	Inhibited	−2.828	8	5.52 e^−12^	11.258
CKAP2L	Inhibited	−4.359	19	5.69 e^−14^	13.245
E2F	Inhibited	−3.256	31	1.06 e^−16^	15.975
TNF	Activated	6.062	137	1.46 e^−11^	10.836
NfkB10Rel	Activated	3.803	15	1.17 e^−11^	10.972
E2F4	Activated	2.401	46	4.18 e^−20^	19.379
CHD1	Activated	3.317	11	3.24 e^−12^	11.489

*Note:* Upstream regulators of differentially regulated pathways after KU757 treatment of A375 and A375MEKi cells.

### 3.3 Identification, Validation and Survival Analysis of Hub Genes

**FIGURE 3:**
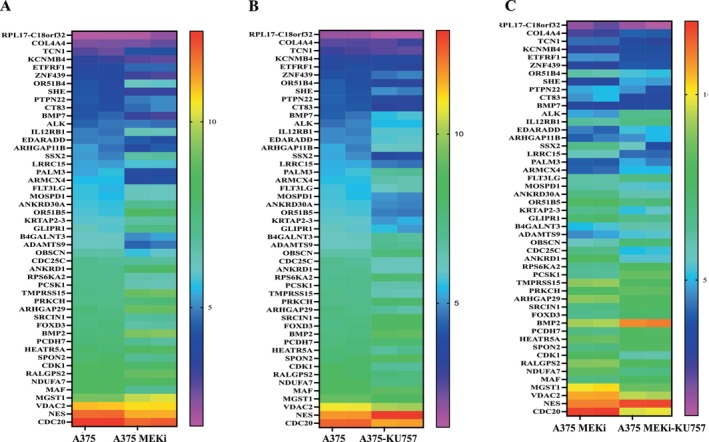
KU757 gene targets in MEK and BRAF resistance. Differentially regulated genes in A375MEKi vs. A375 and inversely regulated after KU757 treatments were considered potential targets for abrogating melanoma resistance pathways. (A) Heat map of mRNA transcripts differentially expressed in A375 versus A375MEKi, and their expression after KU757 treatment in A375 cells (B) or A375MEKi cells (C).

**FIGURE 4:**
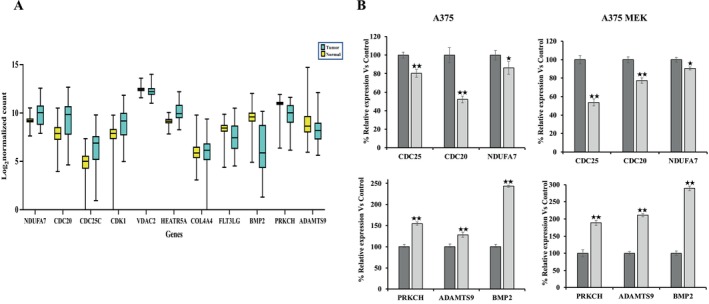
(A) Validation of hub gene mRNA expression levels within TCGA patient sample controls expressed as log_2_ of normalised controls are shown for each gene of interest. (B) RT‐PCR analysis for the validation of hub genes.

**FIGURE 5:**
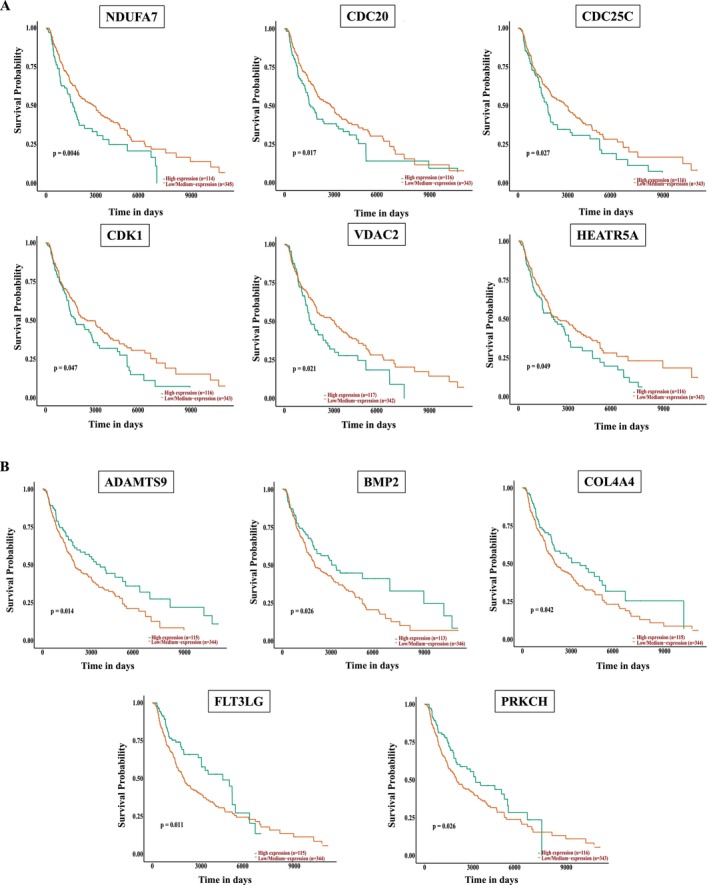
Kaplan Meier Survival Analysis in TCGA melanoma patients stratified by level of hub gene expression. Survival probability charted by time in days. (A) Overall survival was improved with low expression of NDUFA7, CDC20, CDC25C, CDK1, VDAC2 and HEATR5A relative to medium/low mRNA, which was downregulated after K757 treatment. (B) Similarly, overall survival was improved with high expression of ADAMTS9, BMP2, COL4A, FLT3LG and PRKCH, which were upregulated with KU757 treatment.

Hub genes responsible for overcoming MEKi and BRAFi resistance were identified as follows: First, we identified genes that were differentially regulated in the A375MEKi cell line relative to A375 and inversely up‐or down‐regulated in response to treatment with KU757 (Figure 3). From these key targets, STRING network analysis and survival analysis were performed for the identification of hub genes. Together, these data identified NDUFA7, CDC20, CDC25C, CDK1, VDAC2, HEATR5A, COL4A4, FLT3LG, BMP2, PRKCH and ADAMTS9 as hub genes. To validate the expression of hub genes in melanoma relative to normal control samples in the TCGA database XENA database was used (Figure 4A). RT‐PCR analysis (Figure 4B) of melanoma cell lines A375 and A375 MEK after treatment with IC_50_ concentrations of KU757 confirmed the downregulation of CD20, CD25 and NDUFA7, as well as the upregulation of PRKCH, BMP2 and ADAMTS9 hub genes (*p* < 0.05). Finally, correlation of hub genes with overall survival by Kaplan–Meier analysis of Melanoma patients in the TCGA data set was performed with survival stratified by high or low/medium gene expression (Figure 5).

### 3.4 Treatment of Melanoma Cells With KU757 Down‐Regulates Oxidative Phosphorylation, Induces G2/M Cell Cycle Arrest and Apoptosis

**FIGURE 6:**
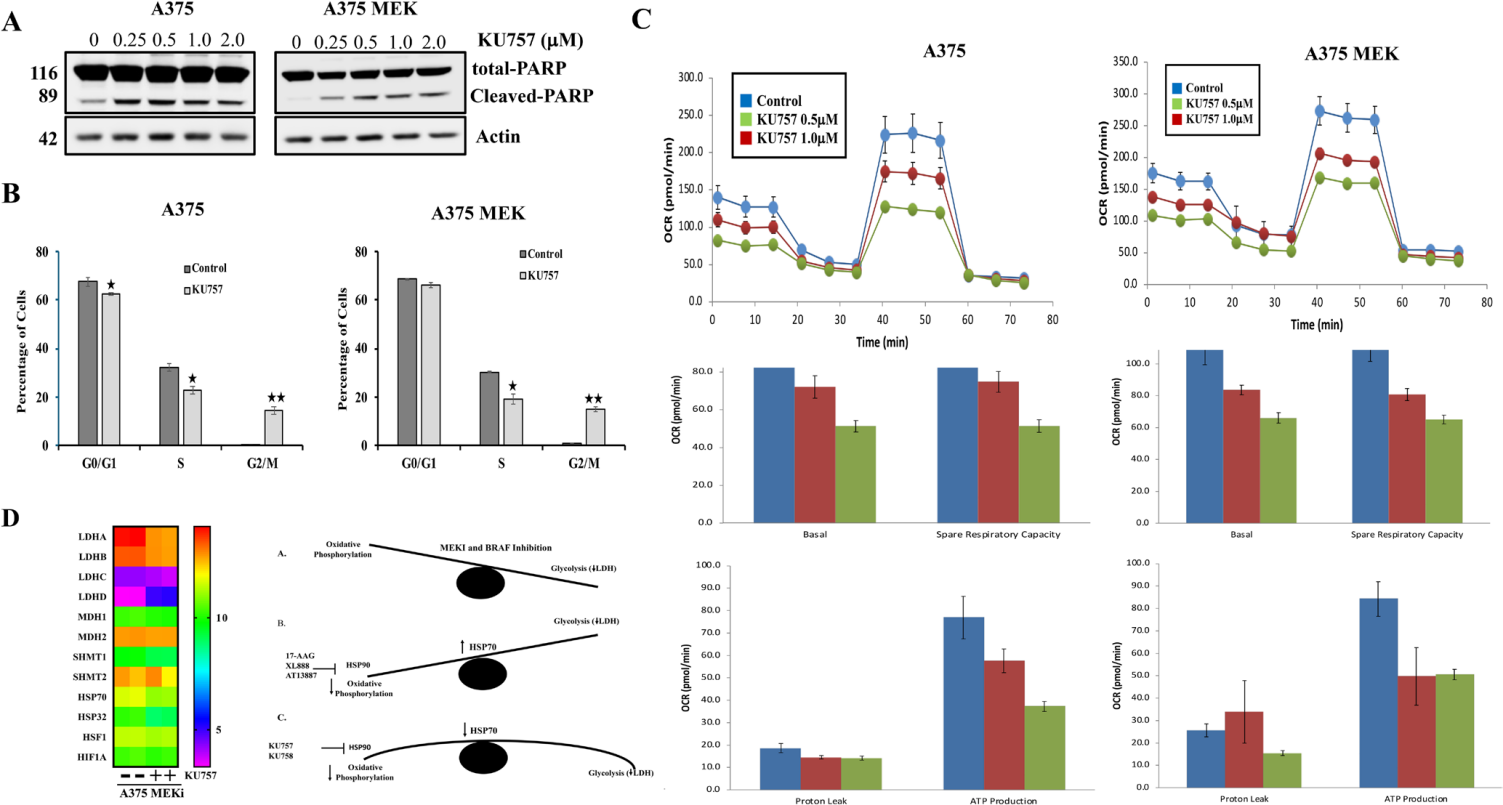
Validation of targeted pathways identified by KEGG analysis after treatment of A375 and A375 MEK cells with KU757. (A) Western blot analysis indicating induction of apoptosis. (B) Cell cycle analysis by flow cytometry. (C) Seahorse analysis of mitochondrial respiration. (D) RNAseq expression in A375MEKi cells of genes involved in glycolysis and the cytoprotective heat shock response with or without KU757 treatment. Expression is downregulated or unchanged after treatment (except for LDHD) suggesting cell metabolism is not dependent on LDH (A‐C), MDH, or SHMT, and the cytoprotective heat shock response is not induced.

To validate the pathways identified through KEGG pathway analysis after treating melanoma cells with KU757, we conducted cell cycle analysis, apoptosis and Seahorse analysis. Dose‐dependent induction of apoptosis was observed after treatment with KU757 for both A375 and A375 MEK, starting from 0.25 μM of KU757 (Figure 6A). Cell cycle analysis of cells treated with the IC_50_ values of KU757 revealed a decrease in the G0/G1 and S phases, which changed from 67.6% and 32.4% to 62.7% and 22.8% for A375, and from 68.7% and 30.35% to 66.1% and 19.1% for A375 MEK. In contrast, the G2/M phase increased from 0.05% to 14.47% for A375 and from 0.92% to 14.78% for A375 MEK, indicating an induction of G2/M cell cycle arrest (Figure 6B). Mitochondrial respiration of melanoma cells treated with KU757 was assessed using the mitostress test in a Seahorse analyzer. The results showed a dose‐dependent decrease in Oxygen Consumption Rate (OCR), as well as reductions in basal and spare respiratory capacity and ATP levels. This indicates that KU757 effectively targets oxidative phosphorylation (OXPHOS) in both A375 and A375 MEK cell lines (Figure 6C).

## 4 Discussion

The first MAPK inhibitors and immunotherapies received FDA approval for use in metastatic melanoma in 2011, and their use has led to a modest response in carefully selected patients [32, 33]. Despite the transient additive benefit of dual MEK and BRAF inhibition, resistance still develops in most patients, creating a need for novel treatment options to prevent and overcome MEKi and BRAFi resistance. The mechanisms of MAPKi resistance in melanoma are multifactorial, and understanding their role is critical to identifying therapeutic targets. Pro‐survival signalling through further upregulation of the MAPK pathway, as well as alternative resistance pathway activation such as PI3K, has been previously established and is the target of many experimental therapuetics [31]. The work presented here indicates that C‐terminal Hsp90 inhibitors demonstrate the ability to not only down‐regulate PI3K/ERK/mTOR signalling and but also are able to dysregulate pathways known to drive MAPK resistance, including pro‐survival signalling pathways, cell cycle regulation and metabolism [34]. Ahn et al. previously demonstrated cellular movement/cancer/cellular response to therapeutics and cell death and survival/cell–cell signalling and interaction/cellular growth and proliferation as the key resistance pathways specific to BRAF resistance in A375 and SK‐MEL‐2 cell lines [35]. KEGG, GO and IPA pathway analysis after treatment of melanoma cell lines (both parent and BRAF/MEK inhibitor‐resistant cell lines) indicated that C‐terminal Hsp90 inhibition with KU757 not only targets pathways associated with cell survival but also other distinct targets, including neutrophil extracellular trap formation, ROS, oxidative phosphorylation and others.

In addition to oxidative phosphorylation, other metabolic pathways that were downregulated after KU757 treatment on GSEA analysis include fatty acid metabolism, peroxisome function and glycolysis. One typical escape mechanism in the setting of inhibited oxidative phosphorylation is the upregulation of glycolytic pathways. Prior studies with N‐terminal Hsp90 inhibitors have shown these compounds are able to shift the metabolic balance toward glycolysis [36]. Cancer cells, especially BRAF mutant melanomas, have been shown to shift toward glycolytic metabolism even in the presence of functional oxidative phosphorylation [37]. This metabolic transition is regulated by both HSF‐1 and Hsp70 [38]. The C‐terminal Hsp90 inhibitors utilised here thus present a unique opportunity to simultaneously downregulate both oxidative phosphorylation and glycolytic metabolism as Hsp70 and HIF‐1 were not upregulated following KU757 treatment (as they normally would be with N‐terminal Hsp90 inhibitors). As metabolic escape pathways are enzyme dependent, we further examined expression levels of lactate dehydrogenase (LDH), methanol dehydrogenase (MDH) and serine hydro methyl transferase (SHMT) and found that mRNA expression was unchanged or downregulated after KU757 treatment except for LDHD (Figure 6D).

To further understand the effect of our C‐terminal Hsp90 inhibitors in targeting upstream regulators of the differentially expressed genes, we utilised Qiagen IPA pathway analysis. The most significant gene down‐regulators with a high degree of directionality included DAP3, ALKBH1, NSUN3 and others. ALKBH1 controls RNA demethylation, which is critical to DNA repair [39]. NSUN3 functions in mitochondrial tRNA modification and dysregulation and has been shown to modulate many key components of tumorigenesis, including cell survival signalling, migration and drug resistance [40]. Lastly, DAP3 is a mitochondrial ribosomal protein, and dysregulation has been shown to be associated with cell death [41]. The most significant upregulated genes after treatment with KU757 include CHD1 and TNF. CHD1 has been shown to be critical to the signalling of DNA damage and has been shown to act as a tumour suppressor in prostate cancer [42]. TNF is known to be an activator of NF‐KB. Its role in cancer can be pro‐survival in the setting of transient expression as it leads to the release of chemokines, metallomatrix proteins and FASL, which abrogates inflammatory signalling. Long‐term expression, however, is known to lead to cell death via necroptosis [43]. Overall, upstream regulator analysis revealed that these genes play an important role in altering several oncogenic pathways targeted by KU757, including DNA repair pathways and cell cycle pathways leading to necroptosis.

To identify hub genes that can be used as a potential biomarkers in melanomas treated with C‐terminal inhibitors, we have also identified those genes differentially regulated by C‐terminal inhibitors relative to their baseline expression in melanoma and that are associated with improved overall survival. Our validation analysis of all identified hub genes revealed ADAMTS9, BMP2, HEATR5A, COL4A4, FLT3LG and PRKCH in UALCAN as targets associated with improved overall survival with upregulation. A disintegrin‐like and metalloprotease with thrombospondin type 1 motif 9 (ADAMTS9) encodes a zinc metalloprotease that has been shown to function as a tumour suppressor in other cancer models leading to downregulation of the AKT/mTOR pathway [44]. Expression is decreased in primary melanoma tumours relative to normal control tissue and it is upregulated following KU757 treatment. Similarly, KU757 leads to upregulation of bone morphogenetic protein 2 (BMP2) which has been shown to have anti‐tumoral effects if associated with BMP‐SMAD pathway activation and TGF‐Beta mediated regulation of cell proliferation and apoptosis [45]. Conversely, the expression of BMP without SMAD leads to further activation of the MAPK pathway. Expression analysis of BMP2 reveals that both primary and metastatic melanomas with poorer survival showed lower expression levels of BMP2, while higher expression was associated with improved survival favouring an anti‐tumoral effect in melanomas. HEATR5A is a paralog of HEATR5B which is associated with binding and membrane trafficking [46]. Its role in cancer has not been clearly defined. COL4A4 encodes type 4 collagen, an important component of the extracellular matrix and basement membrane. Degradation is involved in angiogenesis, remodelling and cancer metastasis [47]. FLT3LG is part of the immune response pathway and is upregulated in the setting of immunogenic cell death [48, 49]. PRKCH is also part of the tumoral immune‐response pathway, complexing with CTLA4 to modulate Treg function [50]. Hence, upregulation of these genes after KU757 treatment would correlate with a more favourable outcome in melanoma.

Five survival‐associated hub genes were downregulated after KU757 treatment, including the cell cycle regulators CDC20, CDC25C and CDK1, as well as mitochondrial function‐associated genes VDAC2 and NDUFA7. Jiang et al. recently also identified CDC20 as a hub gene where high expression was associated with poorer survival in melanoma [51]. CDC25C inhibition is known to function in G2/M cell cycle arrest, acting upstream to regulate CDC1 (which also serves a role in the G2/M checkpoint.) [52] Interestingly, CDC1 upregulation has been shown to act through the SOX pathway in melanoma, which may explain its tumorigenic function despite its typical role in cell cycle regulation [53]. Voltage‐dependent anion channel (VDAC) proteins function as key regulators of mitochondrial metabolism and apoptotic signalling. VDAC2 specifically ensures BAX retro‐translocation to mitochondrial cell membranes, which controls cell fate under apoptosis [54]. As described previously, our analysis strongly indicates inhibition of oxidative phosphorylation, with apoptosis and cell death likely triggered due to the inability to maintain cellular membrane potential, as well as increased reactive oxygen species (ROS) following KU757 treatment. Thus, VDAC2 is an important hub gene that can be a biomarker for the treatment effect of KU757. VDAC downregulation with N‐terminal Hsp90 inhibition had been previously demonstrated, as has subsequent compensatory dependence on alternative metabolic pathways such as glycolysis [55]. This decreased access to oxidative phosphorylation after KU757 treatment is further supported by the downregulation of NDUFA7, a NADH dehydrogenase component which functions in the electron transport chain [56].

In conclusion, these results demonstrate that C‐terminal Hsp90 inhibitors can overcome several chemo‐resistance pathways activated with MEK and BRAF inhibitor resistance in melanomas while simultaneously inhibiting critical pro‐survival pathways, especially those involving the cell cycle and oxidative phosphorylation. Importantly, KU757 downregulated oxidative phosphorylation, without subsequent upregulation of glycolytic pathways. Hsp70 and the cytotoxic heat shock response (a known mediator of this metabolic reprogramming) are not induced with C‐terminal Hsp90 inhibition (as it is with N‐terminal inhibition) supporting further translational analysis of novel C‐terminal Hsp90 inhibitors for use in multidrug‐resistant melanoma. Limitations of this study include that only two melanoma cell lines were tested, which could limit translatability to other melanomas. Also, the mechanism of chemo‐resistance with BRAF and MEK inhibitors is multi‐factorial, and we analysed here a specific group of key resistance pathways in this study. It is quite likely there are other contributors to drug resistance that may not be regulated by Hsp90 chaperone function. As such, expansion of this work to other melanoma cell lines in vitro in addition to translational in vivo evaluation will help define the subpopulation of melanoma patients that would benefit the most from C‐terminal Hsp90 therapy.

## Author Contributions

**Chitra Subramanian:** conceptualization (equal), data curation (equal), formal analysis (equal), investigation (equal), methodology (equal), project administration (equal), supervision (equal), validation (equal), visualization (equal), writing – original draft (equal), writing – review and editing (equal). **Katie K. Hohenberger:** data curation (equal), formal analysis (equal), investigation (equal), methodology (equal), validation (equal), visualization (equal), writing – original draft (equal), writing – review and editing (equal). **Ang Zuo:** resources (equal). **Eric Cousineau:** data curation (equal), validation (equal), visualization (equal). **Brian Blagg:** funding acquisition (equal), writing – original draft (equal). **Mark Cohen:** conceptualization (equal), funding acquisition (equal), project administration (equal), supervision (equal), writing – review and editing (equal).

## Conflicts of Interest

The authors declare no conflicts of interest.

## Supporting information


**Figure S1.** C‐terminal Hsp90 inhibitor chemical structure. (A) Chemical structure of parent compound novobiocin. (B) Backbone structure of KU757 and KU758, where R represent either a hydrogen or methyl side chain.

## Data Availability

The data that support the findings of this study are available on request from the corresponding author. The data are not publicly available due to privacy or ethical restrictions.
