# Supplementary figures and images for: C‐Terminal Hsp90 Inhibitors Overcome MEK and BRAF Inhibitor Resistance in Melanoma
Source: J Cell Mol Med. 2025 Mar 26;29(6):e70489. doi: 10.1111/jcmm.70489 (PMC11937850; doi:10.1111/jcmm.70489)

## Supplemental Figure 1

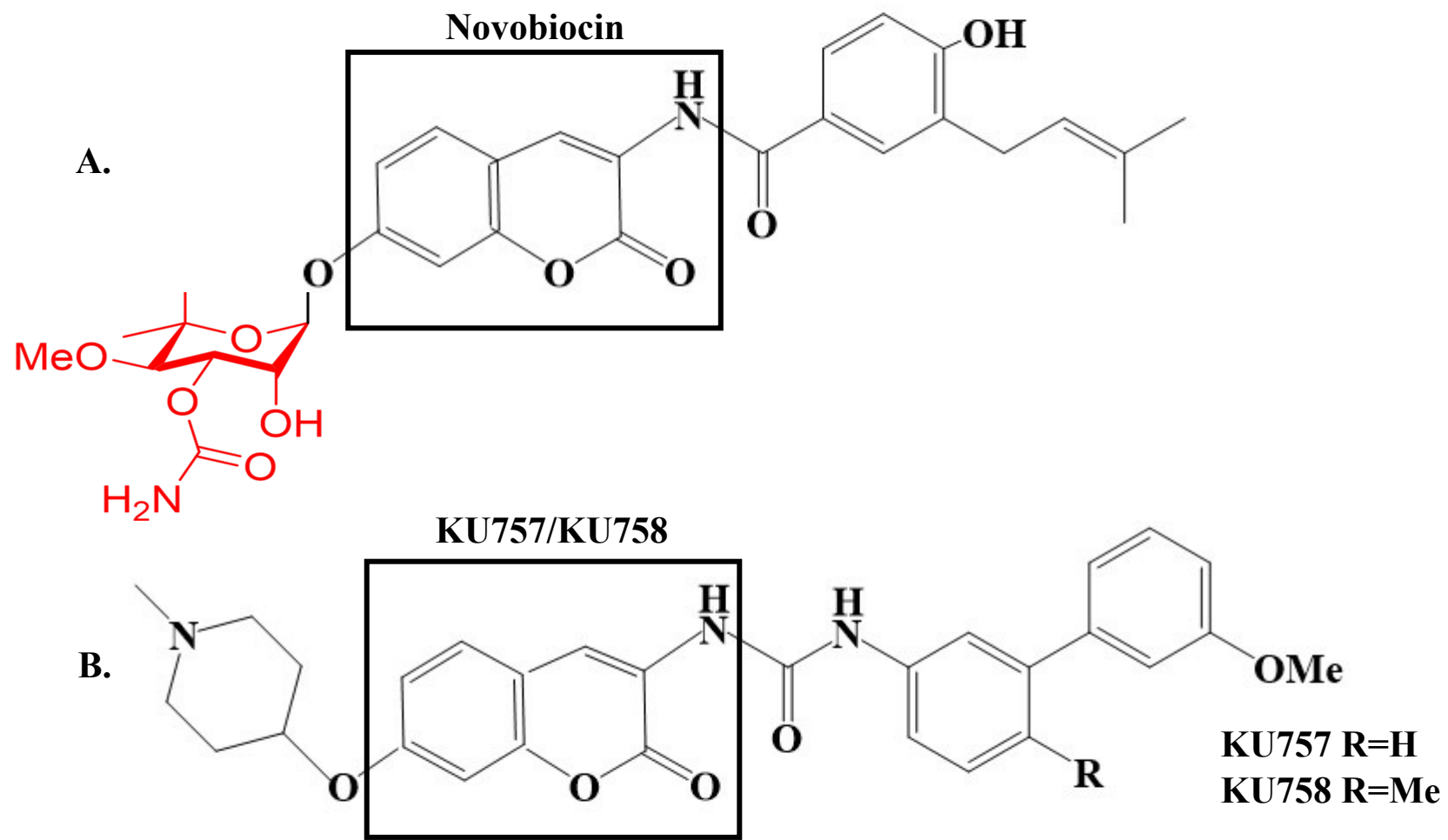

Supplement: Supplementary file 1 — Figure S1. C‐terminal Hsp90 inhibitor chemical structure. (A) Chemical structure of parent compound novobiocin. (B) Backbone structure of KU757 and KU758, where R represent either a hydrogen or methyl side chain. [file JCMM-29-e70489-s001.pdf]
